# microRNAs delivered by small extracellular vesicles in MSCs as an emerging tool for bone regeneration

**DOI:** 10.3389/fbioe.2023.1249860

**Published:** 2023-08-31

**Authors:** Runyuan Liu, Saixuan Wu, Wanqing Liu, Lina Wang, Ming Dong, Weidong Niu

**Affiliations:** School of Stomatology, Dalian Medical University, Dalian, China

**Keywords:** microRNAs, small extracellular vesicles, bone regeneration, mesenchymal stem cells, angiogenesis, osteogenesis

## Abstract

Bone regeneration is a dynamic process that involves angiogenesis and the balance of osteogenesis and osteoclastogenesis. In bone tissue engineering, the transplantation of mesenchymal stem cells (MSCs) is a promising approach to restore bone homeostasis. MSCs, particularly their small extracellular vesicles (sEVs), exert therapeutic effects due to their paracrine capability. Increasing evidence indicates that microRNAs (miRNAs) delivered by sEVs from MSCs (MSCs-sEVs) can alter gene expression in recipient cells and enhance bone regeneration. As an ideal delivery vehicle of miRNAs, MSCs-sEVs combine the high bioavailability and stability of sEVs with osteogenic ability of miRNAs, which can effectively overcome the challenge of low delivery efficiency in miRNA therapy. In this review, we focus on the recent advancements in the use of miRNAs delivered by MSCs-sEVs for bone regeneration and disorders. Additionally, we summarize the changes in miRNA expression in osteogenic-related MSCs-sEVs under different microenvironments.

## 1 Introduction

Bone tissue is a highly vascularized tissue, with abundant vessel networks that transport nutrients and oxygen (Anada et al., 2019). Bone repair after injury occurs in three stages: acute inflammation, bone repair, and remodeling (Claes et al., 2012). However, excessive inflammation negatively affects the osteogenic potential of cells. Therefore, the promotion of bone regeneration relies on angiogenesis, osteogenesis, and anti-inflammatory effect (Bucher et al., 2019).

Mesenchymal stem cells (MSCs) possess the necessary potential for bone regeneration, including homing and multi-lineage differentiation capability (Chen et al., 2021a). However, their transplantation encounters obstacles, such as long-term safety risks (Turinetto et al., 2016; Volarevic et al., 2018; Buduru et al., 2019), tumorigenicity (Miura et al., 2006; Ishihara et al., 2017), cellular senescence (Gong et al., 2020; Luo et al., 2021; Hu M. et al., 2022), and immunological rejection (Zhou et al., 2022a). Therefore, a promising cell-free therapy for bone defect repair and regeneration is extremely urgent. Recent research shown that MSCs stimulate osteogenic differentiation and vasculogenesis through paracrine signaling (Han et al., 2020). Interestingly, the small extracellular vesicles (sEVs) released by MSCs are considered as the executors of this paracrine effect and have potential to replace MSC-based treatments in bone tissue engineering (Hade et al., 2021). Compared to organ transplantation and stem cell therapy, sEVs induce less immunological rejection and provide greater stability for application, transportation, and storage (Liu F. et al., 2019; Kim et al., 2021; Zheng et al., 2021; Tsai et al., 2022). The regenerative functions of MSCs-derived sEVs (MSCs-sEVs) depend on the proteins, lipids, DNA, RNA, and miRNAs they carry. Importantly, miRNAs are associated with bone homeostasis and angiogenesis. However, miRNA-based treatments have encountered challenges due to limited *in vivo* delivery efficiency. Recently, MSCs-sEVs have emerged as a viable tool for delivering therapeutic miRNAs (Zhu et al., 2020). As a natural delivery system, sEVs not only contain abundant miRNAs internally but also serve as carriers, enhancing the stability of encapsulated cargo, prolonging circulation periods, and facilitating transmembrane delivery. However, several challenges persist in the realm of MSCs-sEVs, including the presence of endogenous miRNAs, their potential impact on bone regeneration, and the successful encapsulation of osteogenic miRNAs within sEVs.

**FIGURE 1:**
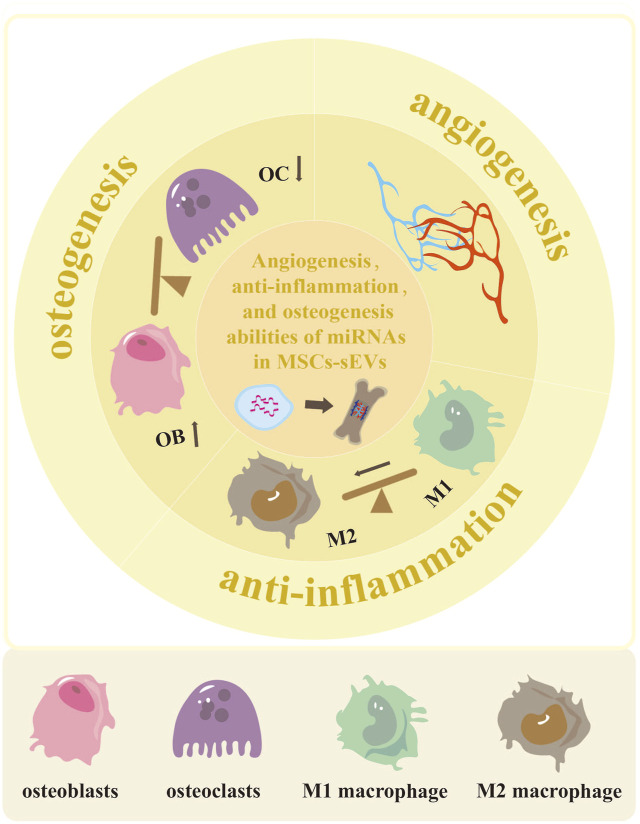
The potential of MSCs-sEVs-miRNAs in enhancing angiogenesis, reducing inflammation, and promoting osteogenesis. The miRNAs derived from MSCs-sEVs exhibit the ability to enhance vascular development and regeneration in cases of bone defects. They also have the potential to promote polarization of M2 macrophage, thus reducing inflammation. Additionally, these miRNAs can facilitate osteoblast differentiation while inhibiting osteoclast differentiation.

This review highlights the delivery of miRNAs by MSCs-sEVs to enhance angiogenesis, reduce inflammation, and promote osteogenesis (Figure 1). To begin with, we provide a concise overview of the biogenesis and functions of sEVs, which serve as nanocarriers for miRNAs. Next, we summarize the alterations in the expression of osteogenic-related miRNAs within MSCs-sEVs, observing diverse microenvironments. Lastly, we discuss the latest research findings concerning the delivery of miRNAs by MSCs-sEVs in the context of bone regeneration and bone-related diseases.

## 2 sEVs as nanocarriers for miRNAs

### 2.1 The biological properties and functions of sEVs

Extracellular vesicles (EVs) are intricate structures with double-layer lipid membrane, ranging in diameter from 40 to 160 nm. They are abundantly found in diverse body fluids being secreted by various cell types including bone mesenchymal stem cells (BMSCs) (Li X. et al., 2020), adipose mesenchymal stem cells (ASCs) (Fang and Liang, 2021), human umbilical cord mesenchymal stem cells (huc-MSCs) (Yang J. et al., 2020), and tumor cells (Kalluri and LeBleu, 2020). EVs can be classified into three distinct groups based on their size: large, medium, and small EVs. Moreover, they can be categorized according to their origin as microvesicles, apoptotic bodies, and exosomes. Microvesicles emerge through cell membrane budding, while apoptotic bodies generate during cell apoptosis (Tkach and Théry, 2016). Exosomes are produced via the formation of intracellular multivesicular bodies (MVBs) that encompass intraluminal vesicles (ILVs). These exosomes are then released through the fusion of MVBs with the plasma membrane and subsequent exocytosis (Gandham et al., 2020). In line with the latest guidelines recommending the use of “sEVs” instead of “exosomes,” this review will adopt the term “sEVs”. The isolation of sEVs can be achieved through various techniques, including differential ultracentrifugation, density gradient centrifugation, microfluidics (Cheng et al., 2021), size exclusion chromatography (Koh et al., 2018), immunoaffinity capture (Stam et al., 2021), or sEVs isolation kits (Macías et al., 2019). To characterize sEVs, their structures, size, and surface markers are examined using transmission electron microscopy (TEM), nanoparticle tracking analysis (NTA), and western blotting (Zhao et al., 2020).

sEVs display diverse physiological functions depending on their origins and contents. Recent research has revealed their crucial roles in various biological processes such as intercellular communication, angiogenesis, tissue regeneration, inflammation, and cancer metastasis (Lv et al., 2018; Ma et al., 2018; Mashouri et al., 2019; Brennan et al., 2020; Kalluri and LeBleu, 2020). Notably, sEVs can regulate the proliferation, differentiation, and apoptosis of target cells through multiple signaling pathways (Vinaiphat and Sze, 2020). For example, ASCs-sEVs activate the SMAD2/3 and SMAD1/5/9 pathways, promoting tendon stem cell proliferation, migration, and tenogenic differentiation (Liu H. et al., 2021). BMSCs-sEVs inhibit apoptosis and inflammation in RAW 264.7 cells via the BRD4/EZH2/TRAIL axis (Su et al., 2021). sEVs derived from dental pulp stem cells (DPSCs) promote cell migration and angiogenic differentiation (Ganesh et al., 2022), while blood serum-derived sEVs facilitate fibroblast migration, angiogenesis, and granulation tissue formation in diabetic mice (Chen et al., 2021b). Hepatocyte-derived sEVs contribute to liver regeneration by increasing sphingosine-1-phosphate synthesis (S1P) (Nojima et al., 2016). MSCs-sEVs alleviate inflammation by suppressing NLRP3 inflammasome activation and the TLR4/NF-κB signaling pathway (Zhang C. et al., 2022). However, carcinoma-associated fibroblasts (CAFs)-derived sEVs increase salivary adenoid cystic carcinoma lung metastasis by inducing lung pre-metastatic niche formation (Kong et al., 2019). Moreover, sEVs play a role in regulating bone metastasis. Prostate cancer-derived sEVs promote the progression of osteolytic lesions in bone metastasis by transferring miR-152–3p from prostate cancer cells to osteoclasts (Ma et al., 2021). Consequently, sEVs derived from various sources exhibit diverse effects, serving as therapeutic agents for tissue regeneration, inflammation suppression, and potential targets for treating cancer metastasis.

sEVs have emerged as valuable tools for both diagnosis and therapy in the treatment of various diseases. Their composition, including proteins, lipids, DNA, RNA, and miRNAs, contributes to their unique properties. sEVs are promising non-invasive diagnostic biomarkers for cancer, diabetes mellitus (Huang et al., 2022), and bone diseases (Li R. et al., 2022). For example, urinary sEVs have been utilized for the early detection of gastric cancer (Chen et al., 2022), hepatocellular carcinoma (Li et al., 2022c), and head and neck cancer (Hofmann et al., 2022). Additionally, the identification of differentially expressed miRNAs within sEVs offers illuminating insights into various processes related to bone diseases such as osteoarthritis, femoral head necrosis, and bone fracture healing (Li et al., 2022b). Consequently, the detection of sEVs has become a simple, non-invasive, highly sensitive, and cost-effective method for monitoring the emergence and progression of diseases.

In the treatment of various diseases, sEVs have demonstrated significant advantages due to their biocompatibility, high bioavailability, and ability to deliver therapeutic cargo to target cells. Stem cell-derived sEVs possess remarkable regenerative potential, making them highly applicable in therapeutic applications. Notably, MSCs-sEVs have been explored as nanotherapeutics for autoimmune and neurodegenerative disorders. MSCs-derived sEVs, for instance, have shown their ability to reduce demyelination and neuroinflammation (Riazifar et al., 2019). Meanwhile, ASCs-derived sEVs have accelerated wound healing by promoting re-epithelialization and reducing inflammation (Zhou et al., 2022b), while BMSCs-derived sEVs have exhibited potential in improving osteoarthritis by promoting cartilage repair and alleviating knee pain (He et al., 2020). Huc-MSCs-derived sEVs have exhibited potential in repairing Parkinson’s disease by crossing the blood-brain barrier (BBB), reducing apoptosis, and preventing the loss of substantia nigra dopaminergic neuron (Chen et al., 2020). sEVs have also been detected in various body fluids. For example, bovine milk-derived sEVs have demonstrated the ability to alleviate colitis symptoms by modulating intestinal inflammatory responses (Han et al., 2022). Plasma-derived sEVs have shown the potential to promote the proliferation and migration of BMSCs while inhibiting inflammation-induced chondrocyte degeneration (Zhang Y. et al., 2022). Furthermore, saliva-derived sEVs have exhibited potential in promoting cutaneous wound healing by stimulating the proliferation, migration, and angiogenesis of human umbilical vein endothelial cells (HUVECs) (Mi et al., 2020). In addition, there is growing interest in using sEVs as delivery tools for therapeutic miRNAs, proteins, and drugs. For instance, miR-31–5p mimics loaded in milk-derived sEVs have demonstrated efficacy in promoting diabetic wound healing (Yan et al., 2022). Zha et al. (2020) reported that encapsulated VEGF plasmid gene within sEVs elevated vascularized osteogenesis *in vivo*. Qian et al. (2022) found that sEVs derived from neural stem cells inhibited glioma by transferring miR-124–3p. Moreover, doxorubicin-loaded neutrophil-derived sEVs have shown potential in the treatment of glioma, brain diseases, and solid tumors (Wang J. et al., 2021). Overall, sEVs possess biocompatibility and exhibit extended circulation time by evading macrophage capture and clearance (Kamerkar et al., 2017). MSCs-derived sEVs offer significant potential in promoting angiogenic and osteogenic differentiation, making them a promising cell-free therapy for bone repair and regeneration (Heris et al., 2022).

### 2.2 sEVs transfer miRNAs into cells

miRNAs are initially transcribed into pre-miRNAs by RNA polymerase II and further processed by Drosha/DGCR8 to generate pri-miRNAs. These pri-miRNAs are exported to the cytoplasm through exportin-5 and mature into functional miRNAs. These mature miRNAs bind to the 3′ untranslated region (3′UTR) of target mRNAs, regulating various physiological and pathological processes through post-transcriptional silencing (Kim et al., 2009). However, the therapeutic potential of miRNAs is hindered by the lack of safe, effective, and stable delivery systems that protect them from degradation and facilitate cellular uptake.

Studies indicated that sEVs contain multiple miRNAs that can be transferred to target cells, influencing their functions. sEVs present potential advantages over other miRNA delivery strategies, including enhanced delivery efficiency and reduced degradation rates (Liang et al., 2021). Factors such as cell source, culture conditions, and sEV isolation techniques can influence the number of miRNAs in sEVs. Loading miRNAs into sEVs can be achieved either by modulating donor cells (endogenously) or by loading cargoes into sEVs *in vitro* (exogenously). Endogenous transfection methods involve modifying source cells to alter miRNA levels (Shojaati et al., 2019; Lou et al., 2020), while exogenous techniques include electroporation, co-incubation, sonication, and lipofectamine for sEVs derived from body fluids like blood, urine, saliva, and breast milk (Asadirad et al., 2019). Notably, the clinical implementation of electroporation (Zhang et al., 2017) for the direct transfer of miRNA mimics or inhibitors into sEVs encounters challenges such as exosome destruction, aggregation, and low loading efficiency (Wei Z. et al., 2021). Exo-Fect transfection has demonstrated high effectiveness, with over 50% transfection efficiency and lower co-localization with lysosomal and early endosomal compartments compared to other methods like heat shock or cholesterol modification of miRNAs (de Abreu et al., 2021). Nevertheless, since sEVs already naturally contain miRNAs and proteins, the efficient encapsulation of additional miRNAs remains unclear. To overcome these challenges and enhance the clinical translation of sEVs for miRNA delivery, further research is needed to optimize their utilization.

## 3 Variations in osteogenic-associated miRNAs expression within MSCs-sEVs under different microenvironments

Altered miRNA expression has been observed in stem cells derived from different sources and cultured under various conditions, as identified through miRNA microarray or high-throughput sequencing techniques. This article provides a summary of effects of osteogenic induction, hypoxic preconditioning, cellular senescence, and chemical or biomaterial microenvironments on the expression of osteogenic-associated miRNAs in MSCs-sEVs.

### 3.1 Osteogenic induction

Despite the potential of MSCs for multi-lineage differentiation, their application in tissue-engineering is limited due to low survival rates and differentiation efficiency. Osteogenic induction medium (OIM) can improve the stability, calcified nodules, and levels of ALP, OCN, OPN, and Runx2 in MSCs. Moreover, it affects the expression of miRNAs in MSCs-derived sEVs. For example, osteogenic induction of huc-MSCs resulted in 67 upregulated and 64 downregulated miRNAs in MSCs-derived sEVs during extended culture. These miRNAs target genes associated with bone growth and function, which are silenced. Notably, the gradually increasing expression of miR-2110 and miR-328–3p promoted osteogenesis by inhibiting the MAPK and PI3K-AKT-mTOR signaling pathways (Yahao and Xinjia, 2021). In BMSCs-sEVs, 8 miRNAs were downregulated, and 16 miRNAs were upregulated under osteoinductive culture, closely linked to bone formation by regulating the balance between Bmpr2/Acvr2b and smad1/5/9 phosphorylation (Liu A. et al., 2021). Moreover, OIM significantly altered miRNA expression over time, with miR-455–3p (Ma et al., 2022) and miR-27a-3p (Ren et al., 2021) continuing to increase on the 7th and 14th days in OIM, resulting in the downregulation of downstream targets HDAC2 and CRY2/ERK1/2. In conclusion, osteogenic induction can alter miRNA expression in sEVs, thereby impacting the osteogenic differentiation ability of sEVs.

### 3.2 Hypoxia preconditioning

Hypoxic pretreatment promotes the viability, proliferation, plasticity, and differentiation of BMSCs, while decreasing their apoptosis via the upregulation of HIF-1α (Luo et al., 2019) and downregulation of stress response-related genes p16 and extracellular signal-regulated kinase (Tsiapalis and Zeugolis, 2019). Recent studies have unveiled the capability of hypoxia preconditioning to elevate miRNA expression in sEVs derived from BMSCs (Shen et al., 2022). For instance, hypoxia preconditioning upregulated miR-126 in MSCs-sEVs via HIF-1α activation. This activation improved bone fracture healing through the miR-126/SPRED1/Ras/Erk signaling pathway (Liu et al., 2020a). Moreover, hypoxic MSCs-derived sEVs stimulate osteogenesis and promote new blood vessel growth in mice with bone deficiencies. During hypoxia, miRNA sequencing analysis demonstrated that elevated levels of miR-210–3p in sEVs. The upregulation of miR-210–3p facilitated vascularized bone regeneration by inhibiting the expression of EFNA3 and activating the PI3K/AKT pathway (Zhuang et al., 2022). Furthermore, MSCs-sEVs derived from the hypoxic preconditioning microenvironment induced the polarization of M1 to M2 phenotype by enriching miR-216a-5p and activating the TLR4/NF-κB/PI3K/AKT axis (Liu et al., 2020b). It is important to note that prolonged periods of hypoxia or excessively low oxygen concentrations may impair the function of MSCs, despite the effectiveness of hypoxic preconditioning in optimizing the regenerative and therapeutic potential of MSCs.

### 3.3 Senescence

Senescence induces irreversible cell-cycle arrest, which in turn contributes to age-related bone fragility and loss. The composition of sEVs and their miRNAs in the bone marrow microenvironment may vary with age. Previous studies have revealed that BMSCs-sEVs from young and aged mice are rich in miRNAs, but the miRNA profile differs significantly, contributing to the dysfunction of stem cells associated with aging. Particularly, aged sEVs exhibited a significant increase in miR-183–5p expression. Transfection of miR-183–5p mimics into BMSCs induced osteoblast dysfunction by downregulating heme oxygenase-1 (Hmox1) activity (Davis et al., 2017). Xu et al. (2018) discovered elevated levels of miR-31a-5p in BMSCs-derived sEVs from aged rats compared to those from their younger counterparts. The enrichment of miR-31a-5p in sEVs regulated osteoblastic and osteoclastic activities, promoting bone resorption and inhibiting bone formation. This observation presents miR-31a-5p as a potential therapeutic modulator for age-related bone loss. Furthermore, sEVs derived from aged bone matrix stimulated adipogenesis and vascular calcification during bone resorption by upregulating the expression of miR-128–3p (Xu et al., 2020), miR-483–5p, and miR-2861 (Wang ZX. et al., 2022). In conclusion, as time progresses, sEVs enriched with specific miRNAs hinder bone formation, enhance bone resorption, and stimulate adipogenesis in bone marrow microenvironment. These molecules hold promise as valuable biomarkers for age-related bone diseases.

### 3.4 Chemical or biomaterials microenvironment

Both chemical elements and biomaterial structures can influence cell-to-cell communication, altering miRNA profiles and target gene expression in sEVs. For example, the incorporation of lithium (Li) into bioactive materials enhanced the proliferation, migration, and tube formation of HUVECs, thereby promoting angiogenesis during bone remodeling. This effect was achieved through the upregulation of miR-130a and activation of the PTEN/AKT signaling pathway in BMSCs-derived sEVs (Liu L. et al., 2019). Similarly, biocompatible titanium alloys supported the attachment of mineralized bone matrix and promoted cell-free bone regeneration by upregulating the expression of miR-146a-5p, miR-503–5p, miR-483–3p and miR-129–5p, while downregulating the expression of miR-32–5p, miR-133a-3p, and miR-204–5p. Coating cell-free titanium alloy scaffolds (Ti-scaffolds) with MSC-sEVs facilitated bone-forming outcomes comparable to those achieved with MSC-seeded Ti-scaffolds (Zhai et al., 2020). In another study, Fe_3_O_4_ nanoparticles were used to manufacture BMSCs-sEV, which were found to promote osteogenesis and angiogenesis by modulating miR-1260a/HDAC7/COL4A2 (Wu et al., 2021a). Similarly, the utilization of strontium (Sr) -containing biomaterials prompted the production of pro-angiogenic miR-146a cargoes within BMSCs-sEVs. These cargoes, in turn, inhibited the expression of Smad4 and NF2, leading to the development of engineered Sr-sEVs with dual-functional regulation for promoting both osteogenesis and angiogenesis in the context of vascularized bone regeneration (Liu L. et al., 2021). Additionally, 3D printing biomaterials loaded with human gingival MSCs-sEVs increased the expression of osteogenic and angiogenic markers such as RUNX2, VEGFA, OPN, and COL1A1, alongside enhanced expression of miR-2861 and miR-210 (Pizzicannella et al., 2019). Taken together, these studies suggest that even tiny bioactive elements or structures can significantly impact the expression of miRNAs in MSCs-sEVs, thereby playing a pivotal role in bone repair and regeneration.

## 4 miRNAs delivered by MSCs-sEVs in bone regeneration

**FIGURE 2:**
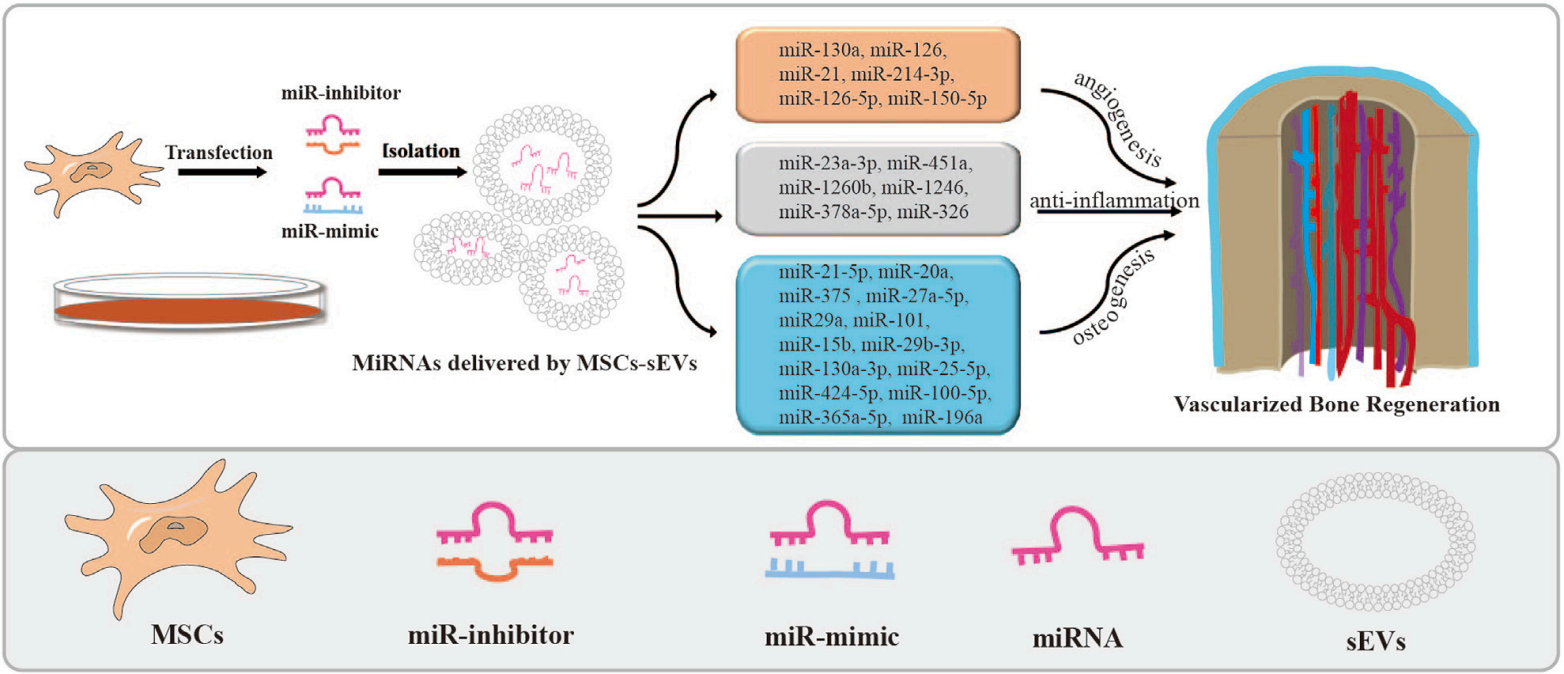
MSCs-sEVs deliver miRNAs by transfection and their applications. The miRNA mimics or inhibitors were transfected into stem cells to overexpress or knock down the expression of miRNAs in MSCs-sEVs. Then, miRNAs loaded in MSCs-sEVs enhance angiogenesis and bone regeneration.

Osteogenesis, angiogenesis, and inflammation are all essential processes involved in the healing of bone defects. miRNAs delivered by MSCs-sEVs and the associated signaling pathways play crucial roles in regulating the above processes (as depicted in Figure 2). These small molecules facilitate intercellular communication, enabling cells to exchange information and participate in the regulation of osteogenesis. Therefore, targeting sEVs-miRNAs and their related signaling pathways holds potential therapeutic opportunities for the treatment of bone injuries and diseases. The formation of blood vessel networks is crucial to supply the necessary nutrients and oxygen required for regulating bone remodeling. The expression of sEVs-miRNAs affects angiogenesis, thereby influencing osteogenesis (Yang et al., 2021). Furthermore, during the inflammatory phase of bone healing, researchers discovered that sEVs-miRNAs enhanced their immunomodulatory properties, leading to the suppression of pro-inflammatory markers and elevation of anti-inflammatory markers (Kang M. et al., 2022). These findings emphasized the importance of the pro-angiogenic and anti-inflammatory abilities of sEVs-miRNAs in promoting bone regeneration.

### 4.1 Facilitating osteoblastic differentiation

Researchers have been investigating the potential application of miRNA mimics or inhibitors to stimulate the proliferation, migration, and differentiation of osteoblasts in stem cells. They have discovered that incorporating these miRNAs into MSCs-sEVs is an effective method for promoting bone formation. For example, BMSCs-sEVs containing miR-21–5p enhanced the differentiation of osteoblasts and increased ALP activity in hFOB1.19 cells (You et al., 2022). Similarly, miR-126–5p and miR-150–5p enriched apical papilla-derived sEVs (SCAP-sEVs) facilitated migration and tube formation of HUVECs, promoted differentiation of MC3T3-E1 cells, and improved bone regeneration (Jing et al., 2022). BMSCs-sEVs were also found to support hBMSCs migration and osteogenesis by transporting miR-20a (Liu et al., 2021d).

These sEVs containing miRNAs operate by binding to the 3′ untranslated region (3′UTR) of downstream targets and mediating various signaling pathways. Compared to BMSCs-sEVs, BMSCs-derived sEVs loaded with miR-21–5p inhibited KLF3, a negative factor in osteogenic differentiation, resulting in enhanced osteoblastic differentiation and ALP activity in target cells (You et al., 2022). BMSCs-sEVs containing miR29a promoted angiogenesis and osteogenesis in a VASH1-dependent manner (Lu et al., 2020). Another research unveiled that sEVs derived from BMSCs, abundant in miR-27a-5p, induced the differentiation of osteoblast cells while suppressing autophagy, leading to bone formation by targeting Atg4B (Li X. et al., 2021). In a calvarial defect model, sEVs derived from miR-375-overexpressing hASCs promoted bone regenerative capacity by binding to insulin-like growth factor binding protein 3 (IGFBP3) (Chen et al., 2019).

MSCs-derived sEVs containing distinct miRNAs can facilitate or inhibit osteogenic differentiation by regulating various signaling pathways. For example, miR-101 encapsulated in BMSCs-sEVs facilitate osteogenic differentiation by repressing FBXW7 to regulate the HIF1α/FOXP3 axis (Li Y. et al., 2021). BMSC-sEVs containing miR-196a targeted Dkk1 to activate Wnt/β-catenin pathway (Peng et al., 2021). Meanwhile, BMSCs-sEVs loaded with miR-140–3p targeted plexin B1 (plxnb1), thereby promoting the osteoblastogenesis function via the plexin B1/RhoA/ROCK signaling pathway (Wang N. et al., 2022). BMSC-sEVs loaded with miR-15b impaired WWP1-mediated KLF2 ubiquitination and inactivated the NF-κB signaling pathway (Li Y. et al., 2020). Moreover, miR-29b-3p encapsulated in BMSCs-sEVs enhanced neovascularization at fracture sites by modulating PTEN/PI3K/AKT (Yang et al., 2022) and KDM5A/SOCS1/NF-κB axes (Zhang et al., 2020b). The overexpression of miR-130a-3p in ASCs-sEVs promoted osteogenic differentiation by mediating SIRT7/Wnt/β-catenin axis (Yang S. et al., 2020). The above studies demonstrate the potential of sEVs-delivered miRNAs in promoting vascularized bone regeneration.

However, it is important to note that some sEVs containing miRNAs have been identified as inhibitors of osteogenesis. For example, the overexpression of miR-25–5p (Lan et al., 2022), miR-424–5p (Wei Y. et al., 2021), and miR-100–5p (Yang et al., 2021) in sEVs inhibited osteogenesis by targeting RUNX2, OCN, ALP, and OPN levels through the SMAD2/ERK, WIF1/Wnt/β-catenin, and BMPR2/Smad1/5/9 signaling pathways, respectively. Huc-MSC-sEVs promoted the proliferation and osteogenesis of BMSCs by suppressing miR-365a-5p via the SAV1/YAP signaling pathway (Kuang et al., 2021). In addition, Long non-coding RNAs (LncRNAs) interact with miRNA and mRNA molecules through the competing endogenous RNA regulatory mechanism in bone repair and regeneration (Yang Y. et al., 2020). Acting as molecular sponges, LncRNAs competitively absorb miRNAs, thereby alleviating their inhibitory impact on target mRNAs and elevating the expression of those specific targets (Liu et al., 2022). For example, overexpressing lncRNA-KCNQ1OT1 in ASCs-sEVs silenced miR-141–5p expression, reducing cytotoxicity and apoptosis of osteoblasts, thus improving osteoporosis (Wang SZ. et al., 2021). Similarly, BMSCs-sEVs containing lnc-H19 absorbed miR-106, thereby promoting osteogenesis through the miR-106-Angpt1-Tie2/NO signaling pathway (Behera et al., 2021). Overall, MSCs-sEVs facilitate osteoblastic differentiation through lncRNA-miRNA-mRNA networks. However, a judicious selection of specific miRNAs is necessary, considering their potential to either promote or inhibit osteogenesis. The main miRNAs-mRNAs networks highlighted in the above studies are summarized in Table 1.

**TABLE 1:** MSC-sEVs delivered miRNAs can regulate many signaling pathways to facilitate osteogenic differentiation.

Stem cells	Delivered miRNAs	Target cells	Target gene	Functions	Signal pathway	Refs
BMSCs	miR-101	MSCs	FBXW7	augment osteogenic differentiation	HIF1α/FOXP3	Li et al. (2021c)
	miR-196a	HFOB1.19	Dkk1	promote osteogenic differentiation	Wnt/β-catenin	Peng et al. (2021)
	miR-140–3p	BMSCs	plxnb1	promote bone formation	plexin B1/RhoA/ROCK	Wang et al. (2022a)
	miR-15b	BMSCs	WWP1	increase ALP activity and osteogenic differentiation-related marker expression	NF-κB	Li et al. (2020c)
	miR-29b-3p	HUVECs	PTEN	enhance neovascularization at the fracture site	PI3K/AKT	Yang et al. (2022)
		BMSCs				
	miR-29b-3p	hFOB1.19	KDM5A	promote angiogenesis and facilitate fracture healing	SOCS1/NF-κB	Zhang et al. (2020b)
	miR-25–5p	BMSCs	SMAD2	promote BMSC osteogenesis	SMAD2/ERK	Lan et al. (2022)
	miR-424–5p	BMSCs	WIF1	attenuate osteogenic development	WIF1/Wnt/β-catenin	Wei et al. (2021a)
	miR-100–5p	HUVECs	BMPR2	inhibit osteogenesis of hBMSCs and angiogenesis of HUVECs	BMPR2/Smad1/5/9	Yang et al. (2021)
		BMSCs				
HUC-MSC	miR-365a-5p	BMSCs	SAV1	promote the proliferation and osteogenesis of BMSCs	SAV1/YAP	Kuang et al. (2021)
ASCs	miR-130a-3p	ADSCs	SIRT7	promote osteogenic differentiation	Wnt/β-catenin	Yang et al. (2020c)

### 4.2 Inhibiting osteoclast differentiation

MSCs-sEVs-miRNAs inhibit osteoclast differentiation, which negatively impacts bone healing by causing bone resorption. For instance, the transfection of miR-27a (Wang Y. et al., 2022) and miR-21 (Hu et al., 2022b) into MSCs increased their expression levels in sEVs, leading to decreased osteoclasts numbers and tartrate-resistant acid phosphatase (Trap) levels through the DKK2/Wnt/β-catenin signaling pathway, ultimately increasing bone mineral density. Similarly, ASCs-sEVs enriched with miR-21–5p and let-7b-5p significantly inhibited osteoclast differentiation, promoted BMSCs migration, and reduced bone resorption (Lee et al., 2021). The abundance of miR-6924–5p in BMSCs-sEVs inhibited osteoclast formation and enhanced bone healing by targeting OCSTAMP and CXCL12 (Feng et al., 2021). Furthermore, gingival tissue-derived MSCs-sEVs-miR-1260b inhibited osteoclastogenic activity by targeting the Wnt5a/RANKL pathway (Nakao et al., 2021). These sEVs-delivery systems exhibit promise for improving bone regeneration by activating osteoblastic differentiation while inhibiting osteoclast differentiation.

### 4.3 Promoting angiogenesis

The growth of blood vessels and the high expression of angiogenic factors are crucial for bone repair (Zhang L. et al., 2020). sEVs play a significant role in promoting vascularized bone regeneration by modulating the expression of miRNAs and multiple signaling pathways. BMSCs-sEVs can promote the expression of proangiogenic cytokines, including VEGF, platelet endothelial cell adhesion molecule-1 (CD31), and HIF-1α, leading to improved vascular development and regeneration in bone defects through the upregulation of the miR-21/NOTCH1/DLL4 signaling axis (Zhang et al., 2021c; Hu H. et al., 2022). The overexpression of miR-130a in BMSCs-sEVs facilitates bone formation and enhances the pro-angiogenic potential of HUVECs by stimulating the PTEN/AKT signaling pathway (Liu L. et al., 2019). The high expression of BMSCs-sEVs-miR-126 enhances angiogenesis by targeting PIK3R2, which activated the PI3K/Akt signaling pathway (Zhang L. et al., 2021). Encapsulation of BMSCs-derived sEVs in scaffolds extends their delivery and released time. sEVs-loaded hydrogels release miR-21, which targets SPRY2 and accelerates both osteogenesis and angiogenesis (Wu et al., 2021b). However, sEVs-miR-214–3p results in impaired angiogenic potential and decreased bone mineral density (Wang X. et al., 2021), highlighting the importance of downregulating this miRNA in sEVs to promote vascularized bone regeneration. Overall, these findings shed light on the regulatory roles of sEVs containing miRNA in angiogenesis during the process of bone remodeling, further emphasizing their potential in bone tissue engineering.

### 4.4 Reducing inflammation

sEVs carrying miRNAs hold significant potential in modulating the bone healing process by regulating the polarization state of host macrophages. The polarization of macrophages plays an essential role in bone healing. M1-type macrophages induce persistent inflammation and tissue degradation, while M2-type macrophages promote anti-inflammatory responses and enhance tissue repair (Mao et al., 2022). The capacity of sEVs to transport and transfer miRNAs renders them a promising avenue for biomedical research. Studies have demonstrated the potential of sEVs in attenuating inflammation and promoting bone tissue repair. For instance, sEVs derived from BMSCs transfected with miR-23a-3p mimics promoted M2 macrophage polarization, reduced inflammation by inhibiting the IRF1 and NF-κB pathways, and improved tendon-bone healing. This was further evidenced by an increased ratio of bone volume to total volume (BV/TV), upregulated collagen type II alpha 1 levels, and improved bone healing outcomes (Li Z. et al., 2022). Similarly, ASCs-sEVs enriched with miR-451a significantly promoted bone healing and facilitated the shift from M1 macrophages to M2 macrophages, thereby inhibiting inflammation via miR-451a/MIF signaling pathway (Li R. et al., 2022). Furthermore, BMSCs-sEVs overexpressing miR-181b facilitated M2 macrophage polarization and osteointegration by suppressing PRKCD while activating p-AKT (Liu et al., 2021e). Additionally, gingival MSCs-derived sEVs containing miR-1260b inhibited osteoclastogenesis and periodontal bone resorption by inducing anti-inflammatory M2 macrophage polarization and repressing Wnt5a/RANKL pathway (Nakao et al., 2021). Dental pulp stem cells derived sEVs (DPSC-sEVs) enhanced T-cell modulatory function, restored the balance between Th17 cells and Treg cells, suppressed inflammation, and accelerated alveolar bone healing by transferring miR-1246 and inhibiting Nfat5 expression (Zhang et al., 2021b). Lastly, huc-MSCs-derived sEVs containing miR-378a-5p (Cai et al., 2021) and miR-326 (Wang et al., 2020) inhibited the expression of interleukin (IL)-18, IL-1β, Caspase-1, and NLRP3 inflammasomes. These findings suggest that miRNAs enriched in MSCs-sEVs possess the capability to regulate macrophage polarization and suppress inflammation, thereby promoting bone regeneration.

## 5 miRNAs delivered by MSCs-sEVs in bone-related diseases

MSCs and their sEVs have shown promising potential as therapeutic agents for bone-related diseases. They can transfer various types of information, regulate immune responses, inhibit cell apoptosis, induce differentiation, and promote tissue regeneration (Malekpour et al., 2022). The miRNAs found in MSCs-sEVs play a crucial role in maintaining the balance between osteoblasts and osteoclasts, promoting angiogenesis, and aiding in bone restoration. Table 2 outlines the important roles of miRNAs from MSCs-sEVs in various bone-related conditions such as bone fractures, osteoporosis, osteoarthritis, and osteonecrosis of the femoral head.

**TABLE 2:** miRNAs delivered by MSCs-sEVs in bone-related diseases.

Bone diseases	Stem cells	Delivered miRNAs	Target cells	Target gene/Signal pathway	Functions	Refs
BF	BMSCs	miR-136–5p	BMSCs, osteoblasts	Wnt/β-catenin	facilitate bone fracture healing	(Hu et al., 2021a; Yu et al., 2021)
		miR-19b		WWP1/Smurf2/KLF5		
		miR-25, miR-29b-3p		SMURF1/Runx2, VapB/Wnt/β-catenin,PTEN/PI3K/AKT		
		miR-335				
OP	BMSCs	miR29a	HUVECs	VASH1	promote angiogenesis and osteogenesis	Lu et al. (2020)
	BMSCs	miR-21–5p	hFOB1.19 cells	KLF3	enhance proliferation and osteoblastic differentiation	You et al. (2022)
	BMSCs	miR-146a	MC3T3-E1 cells	Rtn4/miR-146a	inhibit the viability of cells and promote their apoptosis	Cao et al. (2020)
	BMSCs	miR-34c	hFOB1.19 cells	SATB2	enhance osteoblast activity in osteoporotic mice	Yang et al. (2019)
	BMSCs	miR-186	BMSCs	Hippo signaling pathway	promote osteogenesis in OVX rats	Li et al. (2021a)
	huc-MSCs	miR-1263	BMSCs	Mob1/Hippo	inhibit BMSC apoptosis and prevent OP	Yang et al. (2020a)
	MSCs	miR-27a	Osteoblasts, osteoclasts	DKK2/Wnt/β-catenin	improve bone damage recovery and decrease bone resorption	Wang et al. (2022b)
OA	SMSCs	miR-212–5p	chondrocyte	Runx2	attenuate inflammation, degeneration, and degradation	Wang et al. (2021d), Zheng et al. (2022b)
		miR-155–5p		ELF3		
	BMSCs	miR-125a-5p	chondrocyte	E2F2	accelerate migration and alleviate degradation	Xia et al. (2021)
	BMSCs	miR-206, miR-127–3p, and miR-9-5p	osteoblasts,	Elf3, CDH11, SDC1	ameliorate inflammation and inhibit apoptosis	(Jin et al., 2020; Huang et al., 2021b)
			chondrocyte			
	BMSCs	miR-326	chondrocyte	HDAC3/STAT1/NF-κB p65	inhibit pyroptosis of chondrocytes and cartilage	Xu and Xu (2021)
	BMSCs	miR-122–5p, miR-206	chondrocyte	Sesn2/Nrf2, GIT1	repress autophagy and apoptosis	(Liu et al., 2018; Zhang and Jin, 2022)
ONFH	BMSCs	miR-185–3p	BMSCs	PI3K/Akt, Wnt signaling pathways	regulate osteogenesis-related signaling pathways	Zhu et al. (2022)
		miR-1b-5p				
		miR-129b-5p				
		miR-223–5p				
	BMSCs	miR-224–3p	HUVECs	FIP200	downregulation of miR-224–3p promotes the angiogenesis	Xu et al. (2019)
	CD34^+^ stem cells	miR-26a	HUVECs,	ALP, RUNX2, COL I	increase the bone integrity and vessel density	Zuo et al. (2019)
			BMSCs			
	huc-MSCs,	miR-21–5p	hFOB1.19 cells, HUVECs, BMSCs	SOX5, EZH2	augment angiogenesis and osteogenesis	(Nan et al., 2021; Fang et al., 2022)
	ASCs	miR-378		Sufu, Shh		
	hiPSC-MSCs,	miR-135b	MG-63	PDCD4	inhibit cell apoptosis and promote cell proliferation	(Kuang et al., 2019; Zhang et al., 2020c)
	BMSCs,	miR-150	U-2 cells, osteoblasts	GREM1/NF-κ		
	huc-MSCs	miR-21	MLO-Y4	BPTEN/AKT		

### 5.1 Bone fracture

Bone fracture healing is a complex process, involving the coordinated actions of osteoclasts for bone resorption and osteoblasts for bone formation. Recent studies have highlighted the role of miRNAs encapsulated within BMSCs-sEVs in enhancing neovascularization and bone formation at the fracture site through various complicated signaling pathways. For example, BMSCs-sEVs carrying miR-136–5p promoted osteoblast differentiation and facilitated fracture healing by targeting LRP4 and activating the Wnt/β-catenin signaling pathway (Yu et al., 2021). Another miRNA, miR-19b, abundant in BMSC-sEVs, facilitated bone cell mineralization, and enhanced neovascularization at the fracture site through the WWP1/Smurf2/KLF5/β-catenin signaling pathway (Huang et al., 2021a). Additionally, BMSC-sEVs secrete miR-25 (Jiang et al., 2020), miR-29b-3p (Yang et al., 2022), and miR-335 (Hu H. et al., 2021), which have demonstrated the ability to enhance the proliferation, migration, and differentiation of osteoblasts *in vitro*. *In vivo*, these miRNAs accelerate bone fracture healing through three distinct pathways: SMURF1/Runx2, VapB/Wnt/β-catenin, and PTEN/PI3K/AKT axes, respectively. Collectively, these findings suggest that MSCs-sEVs-encapsulated miRNAs may offer valuable insights into the disappearance of fracture lines, callus formation, and overall fracture healing process.

### 5.2 Osteoporosis

Osteoporosis (OP) is a degenerative bone disease caused by an imbalance in bone remodeling cycle, resulting in an increased risk and susceptibility to bone fractures (Brown, 2017). Recent *in vitro* experiments have demonstrated that certain miRNAs carried by MSCs-sEVs could impact the prognosis of OP. In postmenopausal osteoporotic rats, the expression of BMSCs-sEVs-miR-27a-3p and miR-196b-5p was relatively reduced (Lai et al., 2022), but these miRNAs actually accelerated osteogenesis and reduced bone resorption in OP. Upregulating BMSCs-sEVs containing miR-150–3p (Qiu et al., 2021), miR29a (Lu et al., 2020), and miR-21–5p (You et al., 2022) also stimulate angiogenesis and osteogenesis, presenting a novel therapeutic strategy for treating OP. Conversely, downregulating miR-146a in BMSCs-sEVs enhance the viability of MC3T3-E1 cells and prevent their apoptosis (Cao et al., 2020). BMSCs-derived sEVs containing MALAT1 boost osteoblast activity in osteoporotic mice by inhibiting the expression of miR-34c and promoting the expression of SATB2 (Yang et al., 2019). Mechanistically, miR-186 loaded BMSCs-sEVs facilitate osteogenesis in osteoporotic rats through the Hippo signaling pathway (Li L. et al., 2021). Further research revealed that sEVs derived from huc-MSCs prevent OP by inhibiting BMSCs apoptosis and regulating the miR-1263/Mob1/Hippo signaling pathway (Yang BC. et al., 2020). Overall, MSCs-sEVs carrying specific miRNAs have emerged as a promising therapy for OP, as they promote bone damage recovery and reduce bone resorption through various signaling pathways (Wang Y. et al., 2022).

### 5.3 Osteoarthritis

Osteoarthritis (OA) is a chronic inflammatory disease characterized by the degeneration of chondrocytes, bone sclerosis, and inflammation. Numerous studies have demonstrated the crucial roles of MSCs-sEVs-miRNAs in promoting chondrocyte migration and proliferation (Zhu et al., 2017). In OA tissues, the expression of miR-212–5p and miR-155–5p in synovial mesenchymal stem cells derived sEVs (SMSCs-sEVs) was found to be downregulated. Conversely, upregulated miR-212–5p and miR-155–5p (Wang Z. et al., 2021) in SMSC-sEVs could attenuate inflammation, chondrocyte degeneration, and degradation (Zheng T. et al., 2022). Similarly, MSCs-sEVs enriched with miR-125a-5p exhibited the ability to enhance chondrocyte migration and alleviate extracellular matrix degradation by targeting E2F2 (Xia et al., 2021). BMSC-sEVs encapsulating miR-206, miR-127–3p, and miR-9-5p could ameliorate inflammation and inhibit apoptosis by reducing Elf3 (Huang et al., 2021b), CDH11 (Dong et al., 2021), and SDC1 (Jin et al., 2020), respectively. BMSC-sEVs delivering miR-326 could inhibit chondrocyte pyroptosis and cartilage degradation by targeting HDAC3 through the STAT1/NF-κB p65 axis (Xu and Xu, 2021). Furthermore, recent studies have revealed the involvement of sEVs-lncRNAs in the pathological processes of OA through their interaction with miRNAs. For instance, the lncRNA NEAT1 and lncRNA-KLF3-AS1 delivered by MSCs-sEVs could suppress chondrocyte autophagy and apoptosis and decelerated the progression of OA by modulating the miR-122–5p/Sesn2/Nrf2 (Zhang and Jin, 2022) and miR-206/GIT1 (Liu et al., 2018) axes. In summary, miRNAs and lncRNAs loaded in MSCs-sEVs hold promise in effectively reducing inflammation, alleviating cartilage degradation, and promoting bone regeneration by modulating downstream targets and signaling pathways, thus providing potential therapeutic strategies for the treatment of OA.

### 5.4 Osteonecrosis of the femoral head

Osteonecrosis of the femoral head (ONFH) is a bone disease caused by impaired blood supply and necrosis of the marrow in the femoral head. Recently, studies have shown that the levels of miRNAs in sEVs might be altered during the progression of ONFH (Li et al., 2018). For example, miRNA sequencing revealed decreased expression of miR-185–3p and miR-1b-5p, while miR-129b-5p and miR-223–5p were upregulated in sEVs from femoral tissue in ONFH patients. These changes closely related to classical osteogenesis-related signaling pathways, including PI3K/Akt and Wnt signaling pathways (Zhu et al., 2022). Furthermore, elevated expression of sEVs-miR-100–5p in ONFH inhibits osteogenesis and angiogenesis through the BMPR2/SMAD1/5/9 signaling pathway (Yang et al., 2021). In contrast, in ONFH, the expression of miR-224–3p is downregulated in BMSCs-sEVs, which leads to increased angiogenesis by upregulating FIP200 (Xu et al., 2019). Overall, miRNAs promoting disease progression are upregulated, while those inhibiting disease progression are downregulated in ONFH.

Osteogenesis and angiogenesis are crucial for ONFH treatment, and miRNAs encapsulated within MSC-sEVs play a pivotal role in this process. For example, overexpressing miR-26a in MSCs-sEVs can protect the femoral head by enhancing integrity and density of vessels (Zuo et al., 2019). Recent studies indicated that huc-MSCs-sEVs delivering miR-21–5p promote angiogenesis and osteogenesis by suppressing SOX5 and EZH2 expression (Fang et al., 2022). Other findings suggested that ASCs-sEVs carrying miR-378 accelerate bone regeneration and angiogenesis, inhibiting ONFH progression by targeting Sufu to upregulate the Shh signaling pathway (Nan et al., 2021). Moreover, BMSCs-sEVs delivering miR-148a-3p mimics could enhance BMSCs viability and promote osteogenic differentiation to alleviate ONFH by inhibiting SMURF1 and subsequently increasing SMAD7 and BCL2 expression (Huang S. et al., 2020). Additionally, overexpressing miR-122–5p in sEVs increased osteoblasts proliferation and differentiation in the femoral head via the SPRY2/RTK/Ras/MAPK signaling pathway (Liao et al., 2019).

Osteocyte apoptosis may contribute to bone resorption, trigger osteoporosis, cause microfractures, result in femoral head hypoxia and ischemia, and ultimately lead to ONFH (Xu et al., 2021). Recent reports suggested that sEVs containing miRNAs prevent ONFH by inhibiting osteocyte apoptosis (Li G. et al., 2020). For example, human-induced pluripotent stem cell-derived MSCs-sEVs (hiPSC-MSC-sEVs) could inhibit cell apoptosis, promote cell proliferation, and alleviate the bone loss in ONFH by transferring miR-135b (Zhang et al., 2020c). BMSCs-sEVs delivering miR-150 could alleviate ONFH by suppressing osteoblast apoptosis through the GREM1/NF-κB signal pathway (Zheng LW. et al., 2022). Additionally, sEVs derived from huc-MSCs reduced osteocyte apoptosis in ONFH via the miR-21-PTEN-AKT signaling pathway (Kuang et al., 2019). In conclusion, the miRNAs delivered by MSC-sEVs play a pivotal role in promoting angiogenesis, reducing apoptosis, and facilitating vascularized bone formation in ONFH. This mechanism holds potential as a therapeutic strategy for treating ONFH.

## 6 Conclusion and future perspectives

In summary, bone repair and regeneration rely on the development of bone and blood vessels. MSCs show significant potential for bone regeneration due to their self-renewal and multilineage differentiation capacity (Huang CC. et al., 2020). However, MSCs therapies face obstacles such as high costs, limited sources, and strict storage requirements. Therefore, it is crucial to develop cell-free or acellular approaches to promote bone regeneration.

miRNAs play a crucial role in regulating osteoblast-osteoclast interactions and offer substantial clinical potential (Hu et al., 2022c). However, the lack of effective delivery systems has limited the use of miRNA-based therapeutics. sEVs have emerged as ideal delivery systems due to their ability to maintain miRNA stability during storage. However, sEVs are quickly cleared *in vivo*, which hampers their ability to reach the target site. To overcome this challenge, scaffolds can provide long-term preservation and sustained release of sEVs. For instance, MSCs-sEVs were lyophilized on a microporous bio-glass scaffold, resulting in controlled release, heightened expression of osteogenic-related markers, and enhanced bone repair efficiency (Liu A. et al., 2021). To ensure prolonged retention and controlled release of sEVs, researchers have developed cell-free metal-organic frameworks functionalized with hASCs-sEVs (Kang Y. et al., 2022). These sEVs-loaded composite scaffolds have shown the ability to accelerate blood supply, osteogenic differentiation, and bone reconstruction over an extended period.

However, a major challenge in sEVs research is the impurity and low abundance of conventionally produced sEVs. Three-dimensional (3D) cultures have proven more effective than two-dimensional (2D) cultures in producing sEVs by preventing cell adhesion to culture flask surfaces. sEVs produced from 3D-cultured MSCs exhibit potential in suppressing inflammation and enhancing therapeutic effects in bone regeneration by the upregulation of miRNAs. The upregulation of miRNAs is thought to be caused by the hypoxic conditions in the center of the 3D spheroidal structure (Zhang et al., 2021b). Other findings have shown that combining tangential flow filtration (TFF) with 3D cell cultures can increase the concentration of sEVs in cell culture supernatants, resulting in higher yields of biologically active sEVs and improved transferability of therapeutic siRNAs (Haraszti et al., 2018). Furthermore, the creation of specific sEVs-mimetics (EMs) through sequential mechanical extrusion of cells offers a rapid method for producing large quantities of sEVs, thereby enhancing manufacturing efficiency compared to traditional methods (Zha et al., 2020).

Ultimately, challenges in using sEVs for bone regeneration are their uncertain distribution and lack of targeting ability in the bone microenvironment. Recent research suggested that click chemistry, physical surface modification, and genetic engineering can help sEVs accumulate at the target site and enhance their therapeutic efficacy (Jiang et al., 2022). Several bone-targeting delivery strategies have been developed, including attaching the bone-targeting peptide SDSSD to the membrane of sEVs for specifically delivery to osteoblasts and the promotion of bone formation (Cui et al., 2022). Additionally, C-X-C motif chemokine receptor 4 (CXCR4) positive bone-targeted sEVs could be recruited by BMSCs and released miR-188 to promote osteogenesis and decrease cortical bone porosity for age-related bone loss (Hu Y. et al., 2021). Although the isolation, delivery, and targeted modification of sEVs are relatively well-documented, further research is necessary to understand the mechanisms by which sEVs deliver functional miRNAs to recipient cells. Therefore, it is important to consider good manufacturing practices, stability, loading efficiency, and targeted delivery of sEV-encapsulated miRNAs for bone repair and regeneration.

## Figures and Tables

**Table:** 

**MSCs**	Mesenchymal stem cells
**sEVs**	Small extracellular vesicles
**miRNAs**	microRNAs
**MSCs-sEVs**	MSCs-derived small extracellular vesicles
**BMSCs**	Bone mesenchymal stem cells
**ASCs**	Adipose mesenchymal stem cells
**huc-MSCs**	Human umbilical cord mesenchymal stem cells
**EVs**	Extracellular vesicles
**MVBs**	Multivesicular bodies
**ILVs**	Intraluminal vesicles
**TEM**	Transmission electron microscopy
**NTA**	Nanoparticle tracking analysis
**S1P**	Sphingosine-1-phosphate synthesis
**EZH2**	Enhancer of zeste homolog 2
**TRAIL**	TNF-related apoptosis-inducing ligand
**NLRP3**	NOD-, LRR- and pyrin domain-containing 3
**TLR4**	Toll-like receptor 4
**NF-κB**	Nuclear factor-kappa B
**CAFs**	Carcinoma-associated fibroblasts
**BBB**	Blood-brain barrier
**HUVECs**	Human umbilical vein endothelial cells
**DOX**	Doxorubicin
**DGCR8**	DiGeorge syndrome critical region 8
**3′UTR**	3′ untranslated region
**mRNAs**	Messenger RNAs
**OA**	Osteoarthritis
**OIM**	Osteogenic induction medium
**ALP**	Alkaline phosphatase
**OCN**	Osteocalcin
**OPN**	Osteopontin
**Runx2**	Runt-related transcription factor 2
**PI3K**	Phosphoinositide 3-kinase
**Akt**	Protein kinase B
**mTOR**	Mammalian target of rapamycin
**Bmpr2**	Bone morphogenetic protein receptor type 2
**Acvr2b**	Activin receptor type-2B
**HDAC2**	Histone deacetylase 2
**CRY2**	Cryptochrome 2
**ERK1/2**	Extracellular signal-regulated kinase 1 and 2
**HIF-1α**	Hypoxia-inducible factor 1 alpha
**EFNA3**	Ephrin-A3
**Hmox1**	Heme oxygenase-1
**Li**	Lithium
**Ti**	Titanium
**HDAC7**	Histone deacetylase 7
**COL4A2**	Collagen type IV alpha 2 chain
**NF2**	Neurofibromin 2
**Sr**	Strontium
**CD31**	Platelet endothelial cell adhesion molecule-1
**NOTCH1**	Neurogenic locus notch homolog protein 1
**DLL4**	Delta-like ligand 4
**SPRY2**	Sprouty RTK signaling antagonist 2
**IRF1**	Interferon regulatory factor 1
**BV/TV**	Ratio of bone volume to total volume
**MIF**	Macrophage migration inhibitory factor
**PRKCD**	Protein kinase C delta
**RANKL**	Receptor activator of nuclear factor kappa-B ligand
**DPSC-sEVs**	Dental pulp stem cells derived sEVs
**Nfat5**	Nuclear factor of activated T-cells 5
**IL**	Interleukin
**SCAP-sEVs**	Apical papilla-derived sEVs
**KLF3**	Kruppel-like factor 3
**VASH1**	Vasohibin-1
**Atg4B**	Autophagy-related 4B cysteine peptidase
**IGFBP3**	Insulin-like growth factor binding protein 3
**FBXW7**	Fbox and WD repeat domain-containing 7
**FOXP3**	Forkhead box p3
**KLF2**	Kruppel-like factor 2
**KDM5A**	Lysine demethylase 5A
**SOCS1**	Suppressors of cytokine signaling 1
**SAV1**	Salvador family WW domain-containing protein 1
**YAP**	Yes-associated protein
**NO**	Nitric oxide
**DKK2**	Dickkopf WNT signaling pathway inhibitor 2
**CXCL12**	C-X-C motif chemokine ligand 12
**Trap**	Tartrate-resistant acid phosphatase
**OP**	Osteoporosis
**SATB2**	Special AT-rich sequence-binding protein 2
**Mob1**	Mps one binder kinase activator-like 1
**ONFH**	Osteonecrosis of the femoral head
**SOX5**	SRY-box transcription factor 5
**EZH2**	Enhancer of zeste homolog 2
**RTK**	Receptor tyrosine kinase
**MAPK**	Mitogen-activated protein kinase
**LRP4**	Low-density lipoprotein receptor-related protein 4
**KLF5**	Krüppel-like factor 5
**hiPS**	Human-induced pluripotent stem cell
**VapB**	VAMP-associated protein B
**OA**	Osteoarthritis
**SMSCs**	Synovial mesenchymal stem cells
**CDH11**	Cadherin-11
**SDC1**	Syndecan-1
**HDAC3**	Histone deacetylase 3
**NEAT1**	Nuclear enriched abundant transcript 1
**Nrf2**	Nuclear factor erythroid 2-related factor 2
**3D**	Three-dimensional
**2D**	Two-dimensional
**TFF**	Tangential flow filtration
**EMs**	sEVs-mimetics
**CXCR4**	C-X-C motif chemokine receptor 4
